# Human CD8 T cells generated *in vitro *from hematopoietic stem cells are functionally mature

**DOI:** 10.1186/1471-2172-12-22

**Published:** 2011-03-23

**Authors:** Génève Awong, Elaine Herer, Ross N La Motte-Mohs, Juan Carlos Zúñiga-Pflücker

**Affiliations:** 1 Department of Immunology, University of Toronto, and Sunnybrook Research Institute, 2075 Bayview Avenue, Toronto, Ontario M4N 3M5, Canada; 2 Sunnybrook Health Sciences Centre, Women & Babies Program, 2075 Bayview Avenue, Toronto, Ontario M4N 3M5, Canada

## Abstract

**Background:**

T cell development occurs within the highly specialized thymus. Cytotoxic CD8 T cells are critical in adaptive immunity by targeting virally infected or tumor cells. In this study, we addressed whether functional CD8 T cells can be generated fully *in vitro *using human umbilical cord blood (UCB) hematopoietic stem cells (HSCs) in coculture with OP9-DL1 cells.

**Results:**

HSC/OP9-DL1 cocultures supported the differentiation of CD8 T cells, which were TCR/CD3^hi ^CD27^hi ^CD1a^neg ^and thus phenotypically resembled mature functional CD8 single positive thymocytes. These *in vitro*-generated T cells also appeared to be conventional CD8 cells, as they expressed high levels of *Eomes *and low levels of *Plzf*, albeit not identical to *ex vivo *UCB CD8 T cells. Consistent with the phenotypic and molecular characterization, upon TCR-stimulation, *in vitro*-generated CD8 T cells proliferated, expressed activation markers (MHC-II, CD25, CD38), secreted IFN-γ and expressed Granzyme B, a cytotoxic T-cell effector molecule.

**Conclusion:**

Taken together, the ability to direct human hematopoietic stem cell or T-progenitor cells towards a mature functional phenotype raises the possibility of establishing cell-based treatments for T-immunodeficiencies by rapidly restoring CD8 effector function, thereby mitigating the risks associated with opportunistic infections.

## Background

T lymphocytes develop within the thymus, undergoing distinct differentiation events based on phenotype, while following spatial-temporal cues. The most immature human thymocytes are triple negative for CD3, CD4 and CD8, but express CD34. CD7 is one of the earliest markers to be expressed during human T cell ontogeny, followed by the upregulation of CD1a, which marks T cell commitment. Following this stage, human thymocytes progress to a CD4 immature single positive (CD4ISP) stage, at which point CD4 is expressed in the absence of CD8. Thereafter, a subset of CD4ISP cells are thought to complete TCRβ rearrangement leading to β-selection and differentiation to the CD4^+ ^CD8^+ ^double positive (DP) stage. Finally, following successful TCRα rearrangement, TCRαβ expressing DP thymocytes undergo positive and negative selection events, which result in the production of CD4^+ ^CD8^- ^and CD4^- ^CD8^+ ^single positive (SP) T cells, which then emigrate to the periphery [1].

Cytotoxic CD8 SP T cells play a critical role in adaptive immunity by destroying virally infected or malignant cells. Cytoablative regimens to treat malignancies or HIV infection results in the destruction of T cells, leading to a period of immunodeficiency and a concomitant increase in opportunistic infections [2]. While hematopoietic stem cell (HSC) transplants can reconstitute the entire immune system, T cells are the slowest population to recover or may never recover at all [3,4]. The robust differentiation of multiple sources of HSCs [5-9] into T-progenitors using the OP9-DL1 coculture system suggests that these progenitor cells could be harvested *in vitro *to rapidly restore the T cell compartment *in vivo *and thereby reduce the incidence of opportunistic infection and ultimately improve therapeutic outcomes [10]. While we have demonstrated that specific populations of T-progenitor cells generated *in vitro *can reconstitute the thymus of immunodeficient mice and generate CD8 single positive (SP) T cells *in vivo *[8], it was not clear whether functional maturation of mature CD8 T cells could take place entirely *in vitro *starting from umbilical cord blood (UCB) HSCs in OP9-DL1 cell cocultures.

Mature CD8 T cells were recently reported to be generated following coculture of early thymocyte progenitors (ETPs) on OP9-DL1 monolayers [11]. The absence of a clinically viable source from which to isolate low frequency ETPs, makes it difficult to envision a therapeutic approach to treat T-immunodeficiency based on harvested postnatal thymocytes (PNT). Therefore, it is important to determine whether accessible sources of HSCs, such as umbilical cord blood, directed to differentiate *in vitro *can generate functionally mature CD8 T cells. Two elegant studies have reported the generation of cytotoxic human T cells from HSCs genetically modified to express tumor-specific TCRs cocultured with OP9-DL1 stromal cells [12,13]. Whether *in vitro*-generated CD8 T cells derived from unmanipulated HSCs could also give rise to mature functional T cells and respond to signals delivered through an endogenously generated TCR remained to be tested. Here we demonstrate that long-term coculture of non-transduced human UCB HSCs on OP9-DL1 cells generates αβ-TCR/CD3^+ ^CD8^+ ^SP T cells that exhibit the molecular and cellular signatures corresponding to functionally mature T cells.

## Results

### Phenotypic characterization of human CD8 SP T-cells derived from HSCs cultured with OP9-DL1 cells

We have previously reported the ability to generate CD4^+ ^CD8^+ ^T-lineage cells as well as CD4 and CD8 single positive (SP) cells from human cord blood-derived CD34^+ ^CD38^-/low ^HSC/OP9-DL1 cocultures [7], however the maturational, molecular and functional status of these CD8 SP T-cells were not assessed.

**Figure 1:**
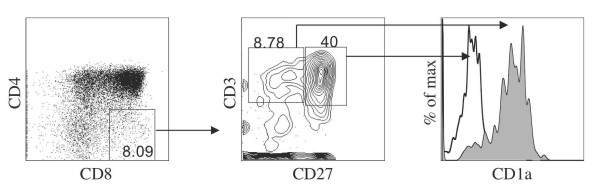
**Characterization of CD8^+ ^T cells generated *in vitro***. Flow cytometric analysis for the expression of CD8 and CD4 from human UCB-derived HSCs cultured on OP9-DL1 cells for 65 days. CD8^+ ^CD4^- ^single positive (SP) cells were gated, as indicated, and analyzed for the expression of CD27 and CD3, with CD1a expression shown for cells gated as CD3^+ ^CD27^- ^or CD27^+ ^(shaded and clear histograms, respectively). Data are representative of over 6 experiments for CD8/CD4 expression and of 3 experiments for CD27/CD1a expression.

To address this, we analyzed long-term cocultures that were allowed to differentiate for an extended period of time. Figure 1 shows the presence of both DP and SP subsets from a day 65 coculture. Examination of multiple cocultures demonstrated the generation of CD8 SP T cells (5.3% ± 1.2% (mean ± SE, n = 6)) after 60-70 days of culture. Additionally, Table 1 displays an analysis for the number of CD8 T cells obtained from individual OP9-DL1 cultures initiated with different numbers of HSCs, which provides an indication of the expected CD8 SP cell yields per input HSC. We further examined the CD8 SP subset present in these cultures for the expression of cell surface markers CD3 and CD27 [14,15], typically expressed on mature T-cells. Amongst the SP CD8 cells (SP8s) found in late cocultures, about 50-60% (Figures 1 for 50% and Figures 3a for 60%) expressed CD3/αβTCR. Of note, the majority (57% ± 10%, n = 3) of CD3^+ ^SP8s were found to co-express CD27. In addition, CD27^+ ^CD3^+ ^CD8^+ ^SPs were also found to lack CD1a expression, which is indicative of functional maturity [16]. This is in contrast to the subset of CD27^- ^CD3^+ ^CD8^+ ^SPs that continued to express CD1a, which is characteristic of the preceding stage in T-cell differentiation [16] and consistent with reports showing that the acquisition of functional maturity by CD3^+ ^thymocytes following positive selection is associated with the expression of CD27 and down-regulation of CD1a expression [15,16].

**Table 1:** Generation of TCR/CD3^+ ^CD8^+ ^cells from CD34^+ ^CD38^-/lo^^w ^hematopoietic progenitors in OP9-DL1 cocultures.

Experiment	**CD34**^**+ **^**CD38**^**-/low **^**(× 10**^**5**^**)**	**TCR/CD3**^**+ **^**CD8**^**+ **^**(× 10**^**5**^**)**^**a**^
1	3.0	0.1
2	8.0	7.0
3	5.0	3.0
4	8.0	3.0
5	1.5	0.4
6	0.3	0.35

^a ^Indicates the number of TCR/CD3^+ ^CD8^+ ^T cells obtained after flow cytometric sort purification from day 60-70 cocultures, which were seeded with the indicated number of CD34^+ ^CD38^-/low ^cells.

### Molecular analysis of in vitro derived human CD8 SP T-cells from UCB HSCs

**Figure 2:**
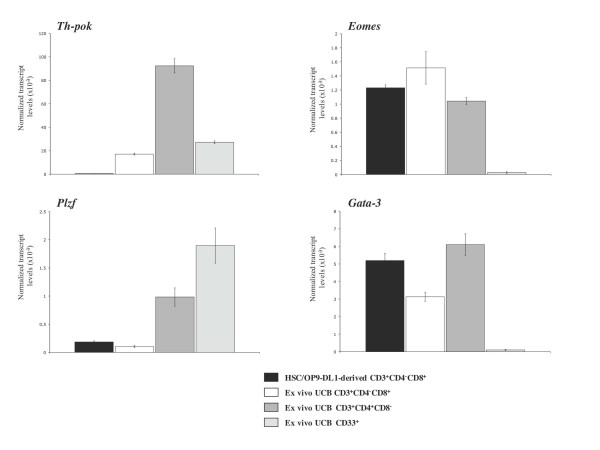
**Gene expression analysis of CD8^+ ^SP T cells obtained from HSCs cultured on OP9-DL1 cells**. Gene expression analysis by QRT-PCR from coculture-derived CD8 SP T cells (CD8^+ ^CD4^- ^CD3^+^). CD8 SP, CD4 SP T cells and CD33^+ ^myeloid cells were purified from the Lin^+ ^fraction of UCB samples and served as controls. Transcript levels for the indicated genes were normalized to human β-actin, and these data are representative of 3 independent experiments, with the standard error bars shown corresponding to values obtained from triplicate wells within an individual experiment.

To gain further insight into the phenotypically characterized CD8 SP T cells derived *in vitro*, we examined the expression of genes known to influence the differentiation or effector status of CD8 T cells by quantitative real-time PCR. We examined the expression of *Th-POK (Zbtb7b)*, *Eomes*, *PLZF (Zbtb16) *and *Gata-3 *transcript levels in SP8 T cells obtained from CD34^+ ^CD38^-/low ^HSCs cocultured with OP9-DL1 cells for approximately 60-70 days. CD4^+ ^CD8^- ^CD3^+ ^(Lin^+ ^SP4), CD4^- ^CD8^+ ^CD3^+ ^(Lin^+ ^SP8) T cells and CD33^+ ^myeloid cells obtained from the mature lineage-positive fraction of UCB were used as controls. Consistent with the role of the zinc finger transcription factor Th-POK in CD4 lineage-commitment [17], *Th-POK *transcripts were barely detectable in coculture-derived SP8 T cells compared to Lin^+ ^SP4 T cells (Figure 2). Also, a low, but detectable, level of *Th*-POK expression was observed in UCB-*ex vivo *Lin^+ ^SP8 T cells. In contrast, the T-box transcription factor *Eomes*, which is a critical regulator for CD8 T cell differentiation and effector function [18], was highly expressed in coculture-derived SP8 T cells at levels that were lower but comparable to that of peripheral SP8 T cells obtained from Lin^+ ^UCB. Interestingly, we also observed a high level of this transcription factor in UCB-derived CD4 T cells, which is consistent with a recent report showing the expression of Eomes in a subset of naïve peripheral CD4 T cells [19]. In addition, the NKT cell/innate T cell transcription factor PLZF [20] was expressed at low levels in HSC/OP9-DL1 coculture-derived CD8 T cells similar to UCB CD8 peripheral T cells. Lastly, we observed high levels of *Gata-3 *transcripts in coculture-derived SP8 T cells, likely owing to the Delta-like-induced signaling of Notch in these cells.

### Generation of functional human CD8 T cells from HSCs cultured with OP9-DL1 cells

**Figure 3:**
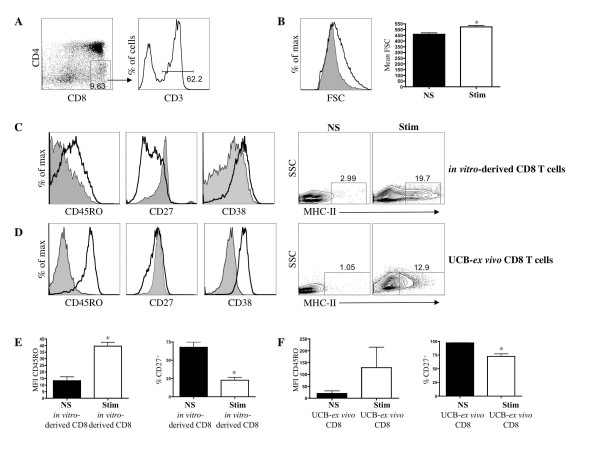
**Characterization of activation marker status on activated CD8^+ ^T cells generated *in vitro***. (A) Day 60-70 HSC/OP9-DL1 coculture-derived CD8 SP T cells were purified as shown, CD8^+ ^CD4^- ^and CD3^+^, and stimulated anti-CD3/CD28 mAbs for 5 days. (B) Left-side panel displays flow cytometric analyses of cell size measured by Forward-light Scatter (FSC) intensity; right-side panel displays mean FSC for non-stimulated (NS) and stimulated (Stim) CD8^+ ^CD3^+ ^cells (n = 5; p = 0.004; t-test). (C) Flow cytometric analyses for the expression of CD45RO, CD27, CD38 and MHC-class II for stimulated (Stim) and non-stimulated (NS) CD8^+ ^CD3^+ ^cells (solid black line and shaded histograms, respectively). (D) Flow cytometric analyses for the expression of CD45RO, CD27, CD38 and MHC-class II for stimulated (Stim) and non-stimulated (NS) CD8^+ ^CD3^+ ^cells obtained from the UCB Lin^+ ^fraction. Analysis of CD45RO mean fluorescence intensity (MFI) and percentage of CD27^+ ^cells for (E) HSC/OP9-DL1 coculture (*in-vitro*)-derived CD8^+ ^CD3^+ ^T cells and (F) UCB-*ex vivo *CD8^+ ^CD3^+ ^T cells were performed (MFI CD45RO *in vitro-derived*, p = 0.02; n = 3; t-test; %CD27^+ ^*in vitro*-derived, p = 0.007; n = 3; t-test; %CD27^+ ^*UCB-ex vivo, *p = 0.03; n = 2; t-test). * indicates statistical significance.

To address the functional status of *in vitro*-generated SP8s, we sorted the CD3/TCR-expressing subset (Figure 3A) and examined whether these cells had the capacity to up/down-regulate downstream differentiation markers, proliferate, express cytolytic effector-function molecules, and secrete γ-interferon (IFNγ) following stimulation with immobilized anti-CD3/anti-CD28 mAbs for 5 days. As shown in Figure 3B, a blast-like appearance based on Forward-light Scatter (FSC) is seen in stimulated (Stim) cells as compared to non-stimulated (NS) cells, which displayed significantly different (*p = 0.004*) FSC mean values when analyzed over multiple experiments (NS, 460 ± 12; Stim, 524 ± 10). Additionally, stimulated cells up-regulated CD45RO, CD38 and MHC-class II expression, and down-regulated CD27 expression, as compared to non-stimulated cells (Figure 3C). As shown in Figure 3D, a similar trend was observed when UCB *ex vivo *CD8 T cells were assayed. However, a more prominent upregulation of CD45RO and CD38, and less prominent downregulation of CD27, was observed. Nevertheless, further analysis of CD45RO MFI and the percentage of CD27 expression (Figure 3E) demonstrated a significant difference between non-stimulated and stimulated *in vitro*-derived CD8 T cells. Indeed, this was also observed for UCB-*ex vivo *CD8 T cells (Figure 3F).

**Figure 4:**
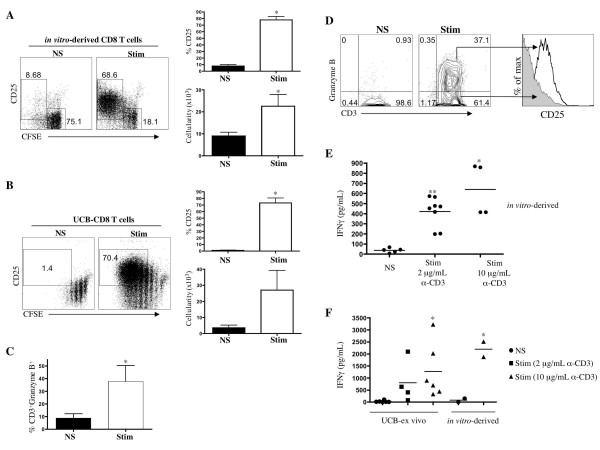
**Functional characterization on activated CD8^+ ^T cells generated *in vitro***. (A) Day 60-70 HSC/OP9-DL1 coculture-derived CD8 SP T cells and (B) UCB-*ex vivo *CD8 T cells were purified and stimulated with anti-CD3/CD28 mAbs for 5 days. Flow cytometric analyses of CFSE levels and CD25 (left side), and percentage of CD25^+ ^cells (upper right) and cellularity (lower right) for stimulated (Stim) versus non-stimulated (NS) are shown (* indicates p < 0.05). (C) Analysis of the percentage of *in vitro*-derived CD8^+ ^CD3^+ ^intracellular Granzyme-B^+ ^expressing cells for stimulated versus non-stimulated is shown (p = 0.02). (E-F) Human IFNγ levels from culture supernatants derived from the above-outlined experiment were determined by ELISA. (E) Statistical significance was measured by unpaired t-test for stimulated and non-stimulated *in vitro*-derived T cells. * (p < 0.005) 2 μg/mL anti-CD3/CD28 stimulated group versus non-stimulated. ** (p < 0.0005) 10 μg/mL anti-CD3/CD28 versus non-stimulated. Data are representative of at least 3 independent experiments, with the exception of the data from the 10 μg/ml stimulations, which are derived from 2 independent experiments. (F) Statistical significance was measured by unpaired t-test for stimulated (10 μg/mL) and non-stimulated UCB-*ex vivo *T cells. * (p < 0.05). Data are representative of at least 4 independent UCB-*ex vivo *CD8 T cells. The data from the *in vitro*-generated T cells (non-stimulated and 10 μg/mL stimulation), are derived from 2 independent experiments.

Furthermore, to address the extent of cellular proliferation induced by TCR-stimulation, sorted CD3^+ ^CD8^+ ^T-cells were loaded with CFSE and incubated for 5 days with or without TCR-stimulation. Figure 4A shows that stimulated cells undergo many rounds of cell division as indicated by the loss of CFSE (left side) compared to non-stimulated cells and by the increase in cellularity (lower right-side). A similar loss of CFSE and increased cellularity was observed in UCB-*ex vivo *T cells (Figure 4B, left-side and lower right-side). Like UCB-*ex vivo *CD8 T cells, we observed that *in vitro-*derived proliferating cells also displayed marked up-regulation of CD25 expression (Figure 4A and 4B, left side). The average percentage of CD25 on the cell surface of stimulated cells was 78% ± 5%, which was significantly higher than on non-stimulated cells (Figure 4A, upper right-side).

To determine whether these TCR-responsive *in vitro*-derived CD8 SP T-lineage cells can be induced to express cytotoxic/effector-function molecules, the expression of Granzyme-B and IFNγ were assessed. Intracellular Granzyme-B expression was detected in approximately 38% of stimulated CD3^+^CD8^+ ^T-cells, as compared to non-stimulated cells that expressed much lower levels of Granzyme-B (Figure 4C). As demonstrated in Figure 4D, when *in vitro*-derived CD8 T cells were gated on Granzyme-B^+ ^cells, these corresponded to CD25^+ ^proliferating cells. Finally, supernatants from wells containing *in vitro*-generated CD8 SPs were analyzed for the presence of IFNγ following stimulation. As shown in Figure 4E, supernatants from cells stimulated with either 2 μg/mL or 10 μg/mL of anti-CD3/anti-CD28 mAbs showed a significant increase in the amount of IFNγ, as compared to non-stimulated cells. In a separate set of experiments (Figure 4F), *in vitro*-derived and UCB*-ex vivo *CD8 T cells were directly compared, and showed similar levels of IFNγ production after TCR/CD28 stimulation. Taken together, these results show that *in vitro*-derived CD8 SP T cells are functionally mature.

**Figure 5:**
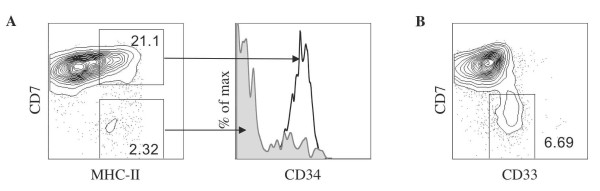
**Characterization of MHC class II-expressing cells derived from OP9-DL1 culture**. Flow cytometric analysis for cell surface expression of (A) MHC class II, CD7 (left side) and CD34 on MHC II^+ ^CD7^+^-gated cells (solid black line) and MHC II^+ ^CD7^-^-gated cells (shaded histogram) from a day 40 HSC/OP9-DL1 coculture, and (B) CD33 and CD7 from a day 45 HSC/OP9-DL1 coculture. Numbers in plots indicate percentage of cells within each gate shown. Data are representative of at least 2 independent experiments.

**Figure 6:**
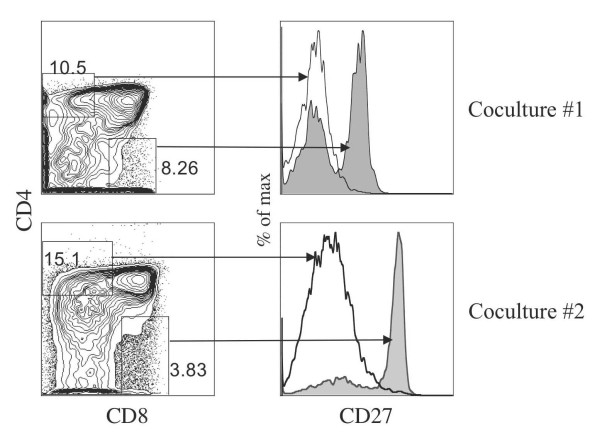
**Characterization of single-positive T cells generated from HSC/OP9-DL1 coculture**. Flow cytometric analysis for cell surface expression of CD4 and CD8 is presented for 2 independent cocultures (left side). CD27 expression was examined on CD4^+ ^CD8^-^-gated cells (solid black line) and on CD4^- ^CD8^+^-gated cells (shaded histogram).

Lastly, we also noted the appearance of CD3^+ ^CD4^+ ^T-cells (Figure 1A and 3A), which could be selected by human MHC class II-expressing cells, which may be a T-lineage cell or a non T-lineage cell such as a dendritic cell. Indeed, when late day cultures were analyzed for the presence of MHC class II expressing cells, MHC-II^+ ^CD34^+ ^CD7^+ ^progenitor T cells and MHC-II^+ ^CD7^- ^non-T-lineage cells were detected as shown in Figure 5A. Figure 5B, demonstrates the generation of CD33^+ ^CD7^- ^myeloid cells, which could provide MHC-II molecules to developing T cells. Interestingly, in contrast to the CD8 SPs, *in vitro*-generated CD4 SP T cells do not show the hallmarks of being functionally mature T-cells. Experiments examining their proliferative ability revealed a lack of responsiveness to CD3 stimulation (data not shown). Also confirming these findings was the lack of CD27 expression on CD4 SP T-lineage cells compared to CD8 T cells in the same culture (Figure 6). Since this molecule is upregulated upon positive selection and present on mature naive T cells, the CD4 SP generated in these cultures may represent transitional cells that require additional differentiation signals that are not readily available in these cultures.

## Discussion

The HSC/OP9-DL1 cocultures not only serve to characterize progenitor function or early events in human T-cell development [8], but may also provide a simple method for the generation of functional human T-cells *in vitro*. This approach may be applicable to cell-based immunotherapies that presently capitalize on T-cell effector-function to induce/enhance anti-tumor eradicating immunity [21]. Thus far, adoptive cell transfer (ACT) for tumor eradication has employed the use of autologous and allogeneic T cells obtained from blood or tumor-infiltrating lymphocytes, followed by expansion protocols to generate millions of cells, with the efficacy of ACT depending on the ability to generate large cell numbers of T cells that maintain effector function. One caveat to stromal-based cultures of HSCs into CD8 T cells is that clinically relevant numbers of CD8^+ ^T cells are not readily obtained (Table 1) following one round of TCR-stimulation. However, one could attempt to overcome this by promoting expansion of CD8 T cells with artificial-APCs and cytokines with several rounds of stimulation [22,23]. Additionally, the number of CD8 T cells obtained in these cultures is directly related to the number of input HSCs; thus, expansion of HSCs prior to differentiation into CD8^+ ^T cells would provide another strategy for the large-scale generation of mature T-lineage cells [24,25]. Nonetheless, we now provide clear evidence for the maturation of functionally responsive CD8 T cells generated from human UCB HSCs in coculture with OP9-DL1 cells.

Although some of the gene transcript levels were not identical to UCB-*ex vivo *CD8 T cells, this may be reflect the fact that coculture-derived T cells are more akin to human post-natal thymocytes, rather than T cells found within the periphery. In addition, some differences in cell surface expression may be due to different temporal kinetics of up/downregulation between *in vitro*-generated versus UCB-*ex vivo *CD8 T cells. Recently, van Coppernolle *et al*. reported the development of CD8 SP αβT cells and large numbers of γδT cells following OP9-DL1 coculture from PNT. While γδT cells can be detected in UCB-derived OP9-DL1 cocultures, we find them at very low frequencies (data not shown). This suggests that the CD34^+ ^PNT, compared to UCB HSCs, have received instructive signals that are Notch independent, which appear to affect specific T-lineage outcomes.

The observation that functionally mature CD8 SP T-lineage cells are generated from HSCs on OP9-DL1 stroma, raises the question of which cell-type, within the cultures, is mediating the MHC-dependent positive selection of this population. It is unlikely that the OP9 cells, which express mouse MHC class I, which is not effectively recognized by human CD8 molecules [26], would supply the required positive selection signals. Rather, a human MHC class I-expressing UCB-derived cell, which may or may not be a T-lineage cell, is likely to be the conveyor of these signals. Although van Coppernolle *et al*. describe the generation of mature CD8 T cells from postnatal thymocytes cocultured on OP9-DL1, and excluded mouse MHC-I as a mode of selection due to its absence on OP9-DL1 cells, our analysis showed that OP9-DL1 cells are in fact MHC class I-expressing cells (data not shown), suggesting that this possibility remains available for supporting CD8 positive selection. Indeed, the high transcript levels of *Eomes, *while low for *PLZF*, suggest that the *in vitro*-derived CD8 SP cells are likely conventional T-lineage cells and not cells with innate-like function and phenotype, which are typically the product of T-to-T selection [27], such as NKT cells, γδ-T cells and innate CD8 T cells [28].

## Conclusions

Taken together, our findings demonstrate that HSC/OP9-DL1 cocultures produce functional CD8 SP T-cells, which are CD3^+ ^CD27^+ ^CD1a^-^, and following TCR-stimulation up-regulate CD25, CD45RO, CD38 and MHC class II, while down-regulating CD27 expression. This complex phenotype is unique to activated human T cells [29,30] and consistent with full effector maturation and effective cytolytic capability [31,32], as seen with the induced expression of Granzyme-B and IFNγ by the *in vitro*-derived CD8 T-cells. The generation of functionally responsive T cells derived *in vitro *from HSCs may broaden the therapeutic avenue for treatment of immunodeficiency and adoptive immunotherapy.

## Methods

### Umbilical cord blood samples

Human umbilical cord blood (UCB) samples were obtained as previously described [8]. Briefly, within 24 hours of collection, cord blood mononuclear cells were isolated using Ficoll density centrifugation and then pre-enriched into lineage (Lin: CD2, CD3, CD14, CD16, CD19, CD24, CD56, CD66b, glycophorin A)-negative (Lin^-^) and lineage-positive (Lin^+^) fractions with the autoMACS-pro cells sorter (Miltenyi Biotec, Auburn, CA) using the StemSep^® ^human progenitor cell enrichment cocktail (Stem Cell technologies, Vancouver, BC, Canada). To isolate the human HSCs, Lin^- ^cells were stained with anti-human CD38-APC and anti-human CD34-FITC mAbs and sorted for CD34^+ ^CD38^-/lo ^cells utilizing a FACSAria cell sorter (BD Biosciences, San Jose, CA). Sorted human HSCs were greater than 99% pure as determined by post-sort analysis.

### Human HSC and OP9-DL1 cell coculture

OP9-control or OP9-DL1 cells were generated and maintained in OP9-media as previously described [5]. Typically, 1-5 × 10^4 ^sorted UCB HSCs (CD34^+ ^CD38^-/lo^) were added per individual well of a 6-well plate containing confluent OP9-DL1 cells, and the cultures were maintained as previously described [33].

### Flow Cytometry

Fluorescein isothiocyanate (FITC)-, R-Phycoerythrin (PE)-, allophycocyanin (APC)-, PE-Cy7-, Alexa Fluor-700 and eFluor 650NC antibodies were purchased commercially (BD Biosciences, San Jose, CA or eBioscience, San Diego, CA). FITC: anti-CD34, anti-HLA-DR/DP/DQ (MHC-II), anti-CD3, anti-CD8; PE: anti-CD27, anti-CD4, anti-CD7, anti-Granzyme B; APC: anti-CD1a, anti-CD25, anti-CD38, anti-CD34; PE-Cy7: anti-CD8; Alexa Fluor-700: anti-CD4; eFluor 650NC: anti-CD3, anti-CD45RO. Intracellular staining for Granzyme B was performed using the Cytofix/Cytoperm kit according to manufacturer's instructions (BD-Biosciences). Cell suspensions were FcRII-blocked and stained, and analyzed with a FACSCalibur (BD-Biosciences) or an LSR-II cytometer. Data analysis was performed using FlowJo software (Tree Star, Ashland, OR) by gating on live lymphocytes and lack of propidium iodide uptake. Numbers in quadrant corners represent percent of gated cells.

### T-cell stimulation assays

UCB-*ex vivo *CD3/TCR-αβ^+ ^CD8^+ ^T cells or *in vitro*-generated CD3/TCR-αβ^+^CD8^+ ^SP cells sorted from HSC/OP9-DL1 cocultures at days 60-70, and 4 × 10^4 ^cells were seeded in individual wells of a flat bottom 96-well plate coated with or without anti-CD3 [Clone UCHT1] (2 or 10 μg/mL) and soluble anti-CD28 [Clone CD28.2] (1 μg/mL) mAbs. All wells contained OP9-media supplemented with rhIL-2 (1 ng/mL) and rhIL-7 (1 ng/mL) (Peprotech, Rocky Hill, NJ or R&D Systems, Minneapolis, MN) cytokines and were analyzed after 5 days. For T-cell proliferation assays, 4 × 10^4 ^*in vitro*-generated CD3^+ ^CD8^+ ^T-cells were sorted and loaded with 10 μM carboxyfluorescein succinimidyl ester (CFSE) according to manufacturer's protocol (Molecular Probes, Eugene, OR) prior to plating. Loss of CFSE labeling was assayed after 5 days of stimulation using a FACSCalibur cytometer or an LSRII cytometer.

### Quantitative real-time reverse-transcriptase polymerase chain reaction (QRT-PCR)

Total RNA was isolated in Trizol-reagent and reverse transcribed using Superscript-III and Oligo(dT)_12-18 _primers (Invitrogen, Burlington, ON). Diluted cDNA samples from sorted coculture-derived CD8^+ ^and from the UCB Lin^+ ^fraction that was sorted for CD4^- ^CD8^+ ^CD3^+ ^T cells, CD4^+ ^CD8^- ^CD3^+ ^T cells and CD33^+ ^cells were used as templates for QRT-PCR reactions. Detection of the QRT-PCR was performed with the SYBR Green PCR master mix according to manufacturer's instructions (Qiagen, Mississauga, ON or Bio-Rad, Hercules CA) on the Applied Biosystems Sequence Detection System 7000 (Life Technologies, Carlsbad, CA). All transcript levels were normalized to human β-actin. Gene-specific forward (F) and reverse (R) primers are as follows: *PLZF*, (F) *AGTGAGTGCAACCGCACCTT *and (R) *GGAAGCAGCTGCCACAGAAC*; *Eomes, *(F) *ATGCAGGGCAACAAAATGTATG *and (R) *GTCTCATCCAGTGGGAACCAGTA*; *Th-Pok, *(F) *GCCTGGACAGCCAAGACAAG *and (R) *AAGGGCTTCTCGCCTGTGT*; *Gata-3, *(F) *GATGGCACGGGACACTACCT *and (R) *GCTCTCCTGGCTGCAGACA*;

## Authors' contributions

GA performed and designed the research, analyzed data, and wrote paper. EH contributed vital reagents. RNLMM performed research. JCZP designed the research, and wrote the paper. All authors read and approved the final manuscript.
